# Isolation, Characterization, and Anti-Biofilm Activity of a Novel *Kaypoctavirus* Against K24 Capsular Type, Multidrug-Resistant *Klebsiella pneumoniae* Clinical Isolates

**DOI:** 10.3390/antibiotics14020157

**Published:** 2025-02-05

**Authors:** Phitchayapak Wintachai, Joanne M. Santini, Renuka Thonguppatham, Maria Stroyakovski, Komwit Surachat, Apichart Atipairin

**Affiliations:** 1 Bacteriophage Laboratory, Walailak University, Thasala, Nakhon Si Thammarat 80161, Thailand; renuka.th@mail.wu.ac.th; 2 School of Science, Walailak University, Thasala, Nakhon Si Thammarat 80161, Thailand; 3 Functional Materials and Nanotechnology Center of Excellence, Walailak University, Thasala, Nakhon Si Thammarat 80161, Thailand; 4 Department of Structural and Molecular Biology, Division of Biosciences, University College London, London WC1E 6BT, UK; j.santini@ucl.ac.uk (J.M.S.); m.stroyakovski@ucl.ac.uk (M.S.); 5 Department of Biomedical Sciences and Biomedical Engineering, Faculty of Medicine, Prince of Songkla University, Hat Yai, Songkhla 90110, Thailand; komwit.s@psu.ac.th; 6 Translational Medicine Research Center, Faculty of Science, Prince of Songkla University, Hat Yai, Songkhla 90110, Thailand; 7 School of Pharmacy, Walailak University, Thasala, Nakhon Si Thammarat 80161, Thailand; apichart.at@mail.wu.ac.th; 8 Drug and Cosmetics Excellence Center, Walailak University, Thasala, Nakhon Si Thammarat 80161, Thailand

**Keywords:** antibacterial activity, bacteriophage, biofilms, K24 capsular type, *Klebsiella pneumoniae*, phage therapy

## Abstract

**Background/Objectives**: The significant outbreak of multidrug-resistant *Klebsiella pneumoniae* has emerged as a primary global concern associated with high morbidity and mortality rates. Certain strains of *K. pneumoniae* are highly resistant to most antibiotics available in clinical practice, exacerbating the challenge of bacterial infections. **Methods**: Phage vB_KpnP_PW7 (vKPPW7) was isolated and characterized. Its morphology, stability, adsorption rate, one-step growth curve, lytic activity, whole-genome sequence analysis, and antibacterial and antibiofilm activities were evaluated. **Results**: The virulent phage has a 73,658 bp linear dsDNA genome and was classified as a new species of the genus *Kaypoctavirus*, subfamily *Enquatrovirinae*, and family *Schitoviridae*. Phage vKPPW7 has a high adsorption rate, a short latent period, and a large burst size. The phage showed activity against 18 *K. pneumoniae* isolates with the K24 capsular type but was unable to lyse *K. pneumoniae* isolates whose capsular type was not classified as K24. Additionally, phage vKPPW7 demonstrated strong stability across various temperatures and pH values. The phage exhibited antibacterial activity, and scanning electron microscopy (SEM) confirmed its ability to lyse MDR *K. pneumoniae* with the K24 capsular type. Furthermore, phage vKPPW7 effectively removed preformed biofilm and prevented biofilm formation, resulting in reduced biofilm biomass and biofilm viability compared to controls. The architecture of phage-treated biofilms was confirmed under SEM. **Conclusions**: These findings suggest that phage vKPPW7 holds promise for development as a therapeutic or biocontrol agent.

## 1. Introduction

*Klebsiella pneumoniae* is a Gram-negative, rod-shaped, facultative anaerobic bacterium commonly found as a typical inhabitant within the nasopharynx and gastrointestinal tract of humans [1]. *K. pneumoniae* poses a significant threat as a human pathogen, causing severe epidemic and endemic nosocomial infections [2]. Outbreaks of *K. pneumoniae* infections have been reported worldwide, leading to various illnesses such as pneumonia, bacteremia, meningitis, and urinary tract infections. Previous studies have associated *K. pneumoniae* bacteremia with a high mortality rate in patients [3,4]. The rapid emergence of antibiotic resistance in *K. pneumoniae*, identified as one of the top three prominent public health threats by the World Health Organization, has raised significant concerns [5,6]. Its global occurrence in recent years has highlighted the urgent need for the development of new antibacterials [7].

A bacteriophage (phage) is a virus that infects and replicates only in bacterial cells, ubiquitous wherever bacteria are found [8,9]. Phage interactions are highly specific to their bacterial hosts, meaning they infect specific bacteria without affecting others. In the case of *K. pneumoniae*, an encapsulated bacterium capable of producing diverse capsular serotypes, *Klebsiella* phages target the capsular polysaccharide (CPS), which is a key determinant of phage specificity [10]. Nowadays, more than 80 *K. pneumoniae* capsular types (K types) have been defined. Phage depolymerase enzymes, which are specific to CPS, degrade the CPS, lipopolysaccharide, or extracellular matrix, thereby allowing the phages to access and bind to the specific receptors on the bacterial membrane. The phages then enter the host cell, replicate, assemble into new virions and use phage-encoded proteins to lyse the bacterial cell, releasing progeny phages. Only virulent phages cause bacterial cell lysis and death [11]. Virulent phages, due to their ability to lyse bacterial cells effectively, are considered potential alternatives or adjuncts to antibiotics for controlling antibiotic-resistant pathogenic bacteria. Various *Klebsiella* phages have demonstrated therapeutic efficacy against *K. pneumoniae*. For example, the *Siphoviridae* phage KP1801 has been shown to reduce extended-spectrum-β-lactamase producing *K. pneumoniae* isolated from clinical samples, both in vitro and *Galleria mellonella*, indicating its therapeutic potential [12]. *Klebsiella* phage vB_KleM_KB2 and phage vB_Kpn_ZC2 showed potential efficacy in the treatment of *K. pneumoniae* infections [13,14]. A phage cocktail composed of 3 *Klebsiella* phages (ɸKpBHU4, ɸKpBHU7, and ɸKpBHU14) reduced colistin-resistant *K. pneumoniae* in a mouse model [15]. In another case, bacteriophages specific to *K. pneumoniae* have been reported. The bacterial load and biofilm formation of *K. pneumoniae* isolated from patients in a mouse model could be reduced by a phage PSKP16 which is specific to capsular serotype K2 [16]. Phage vB_KpnS_Kp13, a member of the *Siphoviridae* family specific to K24 type carbapenem-resistant *K. pneumoniae* isolates, exhibited both antibacterial and antibiofilm activities [17]. The K24 capsular type has been reported to be associated with carbapenemase production and nosocomial infections. Therefore, K24 capsular type has been linked to the spread of pan-drug-resistant strains, posing a significant concern.

Due to the absence of commercially available alternative phage products, this study focuses on isolating and characterizing a *Klebsiella* phage specific to multidrug-resistant (MDR) *K. pneumoniae* with capsular type K24. The phage exhibited high potential for controlling MDR *K. pneumoniae* and possessed antibiofilm activity. Furthermore, the study evaluates the biology of the phage, including its stability, adsorption rate, one-step growth curve, lytic activity, and whole genome sequence analysis.

## 2. Results

### 2.1. Antimicrobial Susceptibility Testing of MDR K. pneumoniae

The antimicrobial susceptibility and resistance profiles of MDR *K. pneumoniae* were assessed against seven antibiotics. All isolates exhibited complete resistance to ampicillin (AMP) and gentamicin (GEN) (Supplementary Table S1). MDR *K. pneumoniae* isolates demonstrated high resistance to cefotaxime (CTX) (90%, 18/20), ceftazidime (CAZ) (90%, 18/20), and ciprofloxacin (CIP) (55%, 11/20). In contrast, all isolates were sensitive to imipenem (IMP) and meropenem (MER). Based on established criteria, all *K. pneumoniae* isolates were classified as MDR, defined as resistance to at least one agent in three or more antimicrobial categories.

### 2.2. Plaque Morphology and Transmission Electron Microscopy (TEM) Characterization of Phage

**Figure 1:**
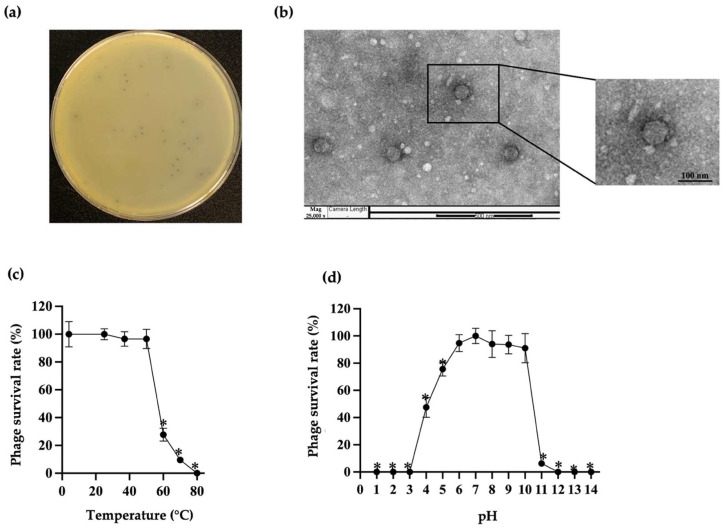
Plaque, virion morphology and stability of phage vKPPW7. (**a**) Representative photographs of phage vKPPW7 plaques formed on a double-layer agar plate; (**b**) Transmission electron micrograph showing the virion morphology of phage vKPPW7; (**c**) Stability of phage vKPPW7 after 2 h incubation at temperatures ranging from 4 to 80 °C; (**d**) Stability of phage vKPPW7 after 2 h incubation at pH levels ranging from 1 to 14. Data are expressed as the mean ± standard error of the mean (SEM), and the * symbol indicates significance at *p* ≤ 0.05.

A *Klebsiella* phage was isolated from an environmental water sample, sourced from Thasala, Thailand, and a purified phage lysate was obtained after three rounds of purification. It was named phage vB_KpnP_PW7, or simply vKPPW7, following the binomial nomenclature for bacteriophages as outlined by the Kropinski guidelines [18,19]. Phage vKPPW7 formed plaques exhibiting a clear center with a diameter of 0.1–0.2 mm and a halo zone with a diameter of 0.2–0.6 mm on a *K. pneumoniae* lawn (Figure 1a). Phage vKPPW7 was observed using TEM, revealing an icosahedral head with a diameter of 68.88 (±2.95) nm and a short tail (Figure 1b). The phage was classified based on morphological criteria into the *Podoviridae* morphology.

### 2.3. Efficiency of Plating (EOP) of Phage vKPPW7

The spot test was performed to assess the efficacy of phage vKPPW7 in eradicating MDR *K. pneumoniae*. This study used 18 clinically isolated MDR *K. pneumoniae* with capsular serotype K24, along with 2 MDR *K. pneumoniae* isolates with non-K24 capsular types. *Acinetobacter baumannii*, Escherichia coli, and methicillin-resistant *Staphylococcus aureus* strains were also included in the spot test. The spot test revealed that phage vKPPW7 could lyse 18 MDR *K. pneumoniae* isolates with the capsular serotype K24. However, phage vKPPW7 did not exhibit infectivity against MDR *K. pneumoniae* isolates with non-K24 capsular types or other bacterial species. Subsequently, the susceptibility of tested isolates to phage vKPPW7 was confirmed through an EOP assay. EOP values are categorized as high (EOP ≥ 0.5), moderate (0.1≤ EOP < 0.5), low (EOP ≤ 0.1), and no activity (EOP < 0.001) based on reproducible infection of the targeted bacteria, relative to phage titer on the isolation strain. The EOP results varied, with nine isolates showing high activity (EOP = 0.63–1), seven showing moderate activity (EOP = 0.12–0.49), and two showing low activity (EOP = 0.05–0.09) (Supplementary Table S2).

### 2.4. Stability of Phage vKPPW7 Under Various Temperatures and pH Values

The thermal and pH tolerance of phage vKPPW7 was evaluated across a range of temperatures and pH values. Phage vKPPW7 exhibited stability within temperatures ranging from 4 to 50 °C. However, a significant decrease in phage viability was observed after incubation at 60 and 70 °C, with a complete loss of viability observed at 80 °C (Figure 1c). Phage viability was also assessed across pH values ranging from 1 to 14. Phage viability remained stable when exposed to pH levels between pH 6 to 10, while a significant reduction in viability was observed at pH levels 4, 5, and 11. Complete loss of phage lytic activity occurred after treatment at pH levels 1, 2, 3, 12, 13, and 14 (Figure 1d).

### 2.5. Phage Adsorption

**Figure 2:**
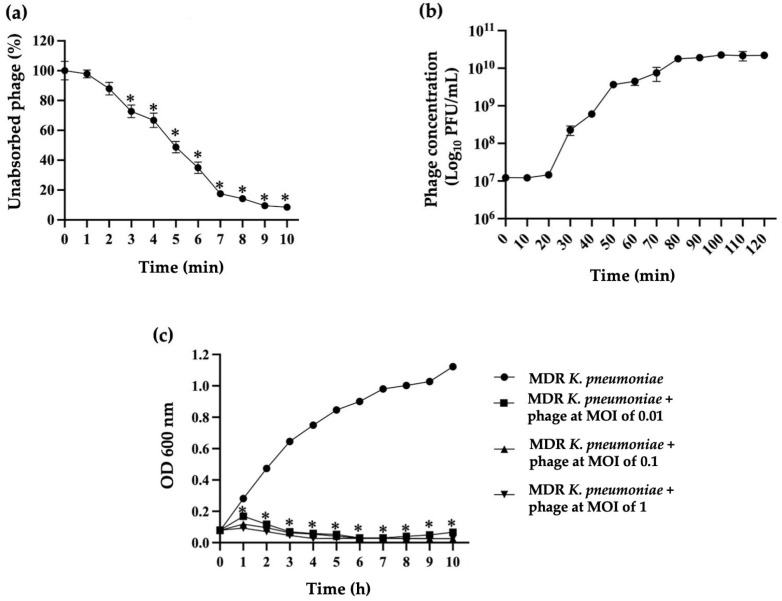
Infective properties of phage vKPPW7. (**a**) Adsorption curve of phage vKPPW7 to the MDR *K. pneumoniae* clinical isolate KPPW67, which served as the host cells; (**b**) One-step growth curve showing latent period and burst size of phage vKPPW7 on its host bacteria; (**c**) The lytic activity of phage vKPPW7 against its host bacteria at different multiplicities of infection. The bars represent the SEM, and asterisks denote statistically significant differences (* *p* ≤ 0.05).

The adsorption rate of phage vKPPW7 onto MDR *K. pneumoniae* was quantified. Within 7 min after the phage and bacteria were mixed, approximately 80% of the phage was adsorbed. By 10 min post-incubation, the phage adsorption rate exceeded 90% (Figure 2a). The adsorption rate constant (*k*) of phage vKPPW7 was 2.46 × 10^−9^ mL/min.

### 2.6. One-Step Growth Curve of Phage vKPPW7

A one-step growth curve of phage vKPPW7 against the host strain was conducted. The infection of phage vKPPW7 exhibited a latent period, defined as the time from the phage adsorption on the bacterial cell to the release of new phage, of approximately 20 min (Figure 2b). A rapid increase in phage titer was detected at 50 min post-co-cultivation, reaching a plateau at 80 min. The burst size of the phage, calculated as the ratio of the final phage concentration to the number of initially infected bacteria, was approximately 106 plaque-forming units per cell (PFU/cell).

### 2.7. In Vitro Antibacterial Activity of Phage vKPPW7

The efficacy of phage vKPPW7 in inhibiting the growth of MDR *K. pneumoniae* was evaluated in vitro. MDR *K. pneumoniae* was infected with the phage at the multiplicity of infections (MOIs) of 0.01, 0.1, and 1, and bacterial growth (as OD600) was measured over the course of 10 h by a spectrophotometer. The absorbance of bacterial growth without phage infection was used as a control (Figure 2c). The absorbance of MDR *K. pneumoniae* infected with phage vKPPW7 at MOIs of 0.01, 0.1, and 1 was significantly lower than that of the control at 1 h post-infection. At the 2 h mark of the infection period, the absorbance of phage-infected MDR *K. pneumoniae* had gradually reduced. However, after 8 h incubation, the absorbance of MDR *K. pneumoniae* infected with phage vKPPW7 at an MOI of 0.01 began to increase, while that of MDR *K. pneumoniae* infected with phage vKPPW7 at MOIs of 0.1 and 1 continued to decrease.

### 2.8. Morphological Changes in Bacterial Cells Following Phage Infection

**Figure 3:**
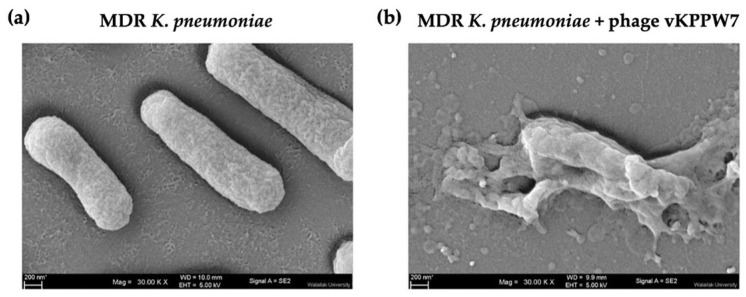
Structural changes in MDR *K. pneumoniae* following treatment of phage vKPPW7. (**a**) MDR *K. pneumoniae* bacterial cells; (**b**) MDR *K. pneumoniae* cells treated with phage vKPPW7.

The effect of phage vKPPW7 infection on the morphology of MDR *K. pneumoniae* cells was observed under a scanning electron microscope (SEM). In the control group, the bacterial cells maintained their normal shape, and the bacterial membrane appeared smooth and intact (Figure 3a). Conversely, in the case of bacterial cells infected with the phage, the membrane was visibly ruptured, indicative of bacterial lysis and consequent cell death. Phage particles were observed on the ruptured bacterial cell membrane, and substantial debris was observed on the coverslip (Figure 3b).

### 2.9. Whole Genome Sequencing and Bioinformatics Analysis

**Figure 4:**
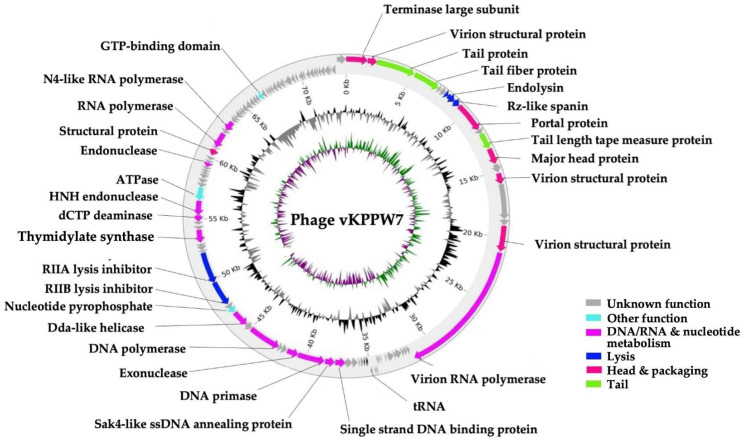
Genome map of phage vKPPW7. The innermost circles depicted the GC skew and GC content, respectively. Arrows represented the predicted coding sequences (CDSs), with their colors indicating the grouping of CDSs based on the protein functions.

The genome of phage vKPPW7 was sequenced using the Illumina sequencing platform, followed by de novo assembly and annotation. The genome of phage vKPPW7 is 73,653 bp in length, with a G + C content of 44.12% (Figure 4). The phage genome was annotated by the Pharokka program (Supplementary Table S3). The phage genome contained 109 predicted coding sequences (CDSs), with 31 assigned functional annotations, 78 unknown functions, and 4 tRNA genes. The functional annotations were classified into 4 main functions: head and packaging (including terminase large subunit, 3 virion structural proteins, portal protein, major head protein, and structural protein with Ig domain), tail (tail length tape measure protein and tail protein), DNA, RNA and nucleotide metabolism (RNA polymerase, N4-like RNA polymerase, virion RNA polymerase, single strand DNA binding protein, Sak4-like ssDNA annealing protein, DNA primase, DNA polymerase, Dda-like helicase, thymidylate synthase, dCTP deaminase, HNH endonuclease, and endonuclease), lysis (holin, endolysin, R2-like protein, RIIA lysis inhibitor, RIIB lysis inhibitor) and other (nucleotide pyrophosphohydrolase, and ATPase). Given the high number of CDSs with unknown functions, the phage genome was further annotated using the FASTEST program. The genome encoded 89 predicted genes, and protein prediction results identified 89 putative proteins, with 44 proteins predicted to be functional proteins of phages and 45 hypothetical proteins (Supplementary Table S4). These gene products were categorized into three primary clusters: DNA replication/modification, structural proteins, and host lysis components. The DNA replication/modification cluster included proteins such as large subunit terminase, small subunit terminase, RNA polymerase I subunit, 2 putative helical domains, putative DNA processing chain, antirepressor protein, 2 putative transmembrane helical domain, ATPase, metallopeptidase, deoxycytidine triphosphate deaminase, thymidylate synthase, rIIA-like protein, rIIB-like protein, triphosphate pyrophosphohydrolase, DNA helicase, DNA polymerase I, 3′-phosphatase 5′-polynucleotide kinase, nuclease superfamily protein, DNA primase, nucleoside triphosphate hydrolase, single-stranded DNA-binding protein, holliday junction resolvase, virion RNA polymerase, putative four transmembrane helical domains containing protein, and N-acetylmuramidase. The structural proteins cluster featured tail spike protein, tail fiber protein, tail protein, capsid decorating protein, 2 structural proteins, putative tail protein, major coat protein, tape-measure protein, portal protein, and tail length tape-measure protein. The host lysis components cluster comprised putative peptidoglycan binding domain, Rz/RzI spanin, and holin. Additionally, a depolymerase domain was identified on the tail fiber of phage vKPPW7 using Phyre 2.

**Figure 5:**
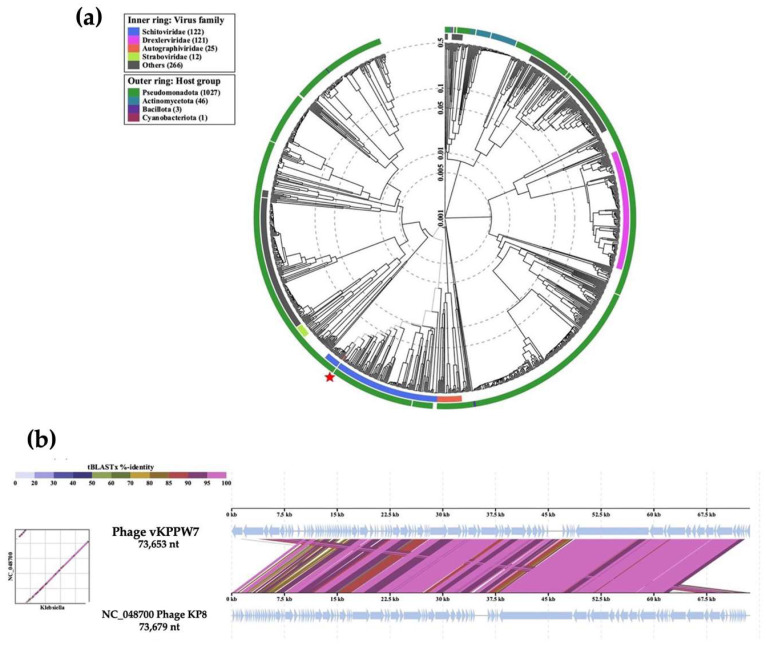
Phage vKPPW7 genomic analysis. (**a**) A phylogenetic tree of phage vKPPW7 and related phages was generated using ViPTree, a whole-genome-based phylogenomic tool. The query sequence is indicated by an asterisk.; (**b**) A genome-wide comparison of phage vKPPW7 and *Klebsiella* phage KP8 was performed using ViPTree. Homologous regions identified by tBLASTX search are connected by segments, which are colored-coded based on amino acid identity.

No bacterial proteins and CRISPR elements were found within the genome. A phylogenetic tree was constructed using the ViPTree, a whole-genome-based phylogenomic tool. The data was analyzed based on genome-wide sequence. The results revealed that phage vKPPW7 is closely related to *Klebsiella* phage KP8 (NC_048700.1), which targets *K. pneumoniae* strains outside the K1 and K2 capsular types [20]. This indicates that phage vKPPW7 belongs to the genus *Kaypoctavirus*, subfamily *Enquatrovirinae*, family *Schitoviridae*, and order *Caudoviricetes* (Figure 5a). The taxonomy of the phage was confirmed using the taxmyPHAGE program.

The whole genome sequence alignment between phage vKPPW7 and *Klebsiella* phage KP8 was analyzed using the ViPTree (Figure 5b). The alignment showed regions of both high and low similarity. The overall percent identity of the genomes was calculated using EMBOSS Stretcher to assess the pairwise alignment of nucleotide sequences [21]. The results revealed that phage vKPPW7 exhibited 66.9% DNA sequence identity with *Klebsiella* phage KP8.

### 2.10. Evolutionary Position Within the Schitoviridae

**Figure 6:**
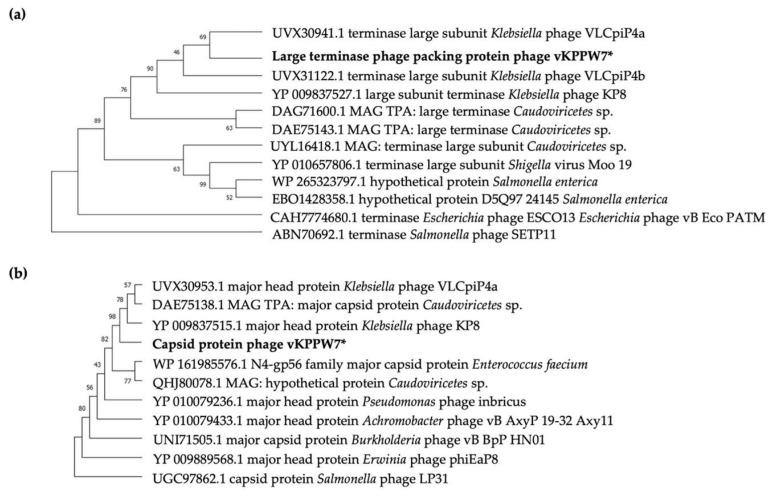
Evolutionary relationship between phage vKPPW7 and other phages. The phylogenetic trees for phage terminase large subunit (**a**) and capsid proteins (**b**) were constructed using the maximum likelihood method based on the alignment of amino acid sequences. The JTT matrix-based model was utilized with 1000 bootstrap replicates. The sequence of phage vKPPW7, used as the query, is indicated by an asterisk (*).

To investigate the evolutionary relationship of phage vKPPW7 proteins with related phages, the large terminase, a DNA packing protein, and the capsid, a structure-related protein, were used as models. Analysis of amino acid sequences of the large terminase, a highly specific protein responsible for DNA recognition and initiation of DNA packaging, revealed that the sequences of phage vKPPW7 shared a high identity (99.81%) with the terminase large subunit of *Klebsiella* phage VLCpiP4a (UVX30941.1). Furthermore, the large terminase of phage vKPPW7 shared a clade ancestor with the *Klebsiella* phage VLCpiP4b (UVX31122.1, 99.44% identity) (Figure 6a). Additionally, they clustered to the same clade with the large subunit terminase of *Klebsiella* phage KP8 (YP_009837527.1, 99.44% identity).

The capsid, another crucial protein for studying the evolutionary history of phages [22], exhibited a close relationship between phage vKPPW7 and other phages upon alignment of the capsid protein sequence with those of closely related phages. The capsid of phage vKPPW7 demonstrated a high degree of similarity to the major head protein of *Klebsiella* phage KP8 (YP_009837515.1, 98.75% identity) (Figure 6b). Moreover, the capsid of phage vKPPW7 was clustered to the same clade with the major capsid protein of a phage in Realm *Duplodnaviria*, Kingdom *Heunggongvirae*, Phylum *Uroviricota* of the class *Caudoviricetes* (DAE75138.1, 98.5% identity), and the major head protein of *Klebsiella* phage VLCpiP4a (UVX30953.1, 98.5% identity). These findings indicate a close evolutionary relationship between phage vKPPW7 and members of the *Kaypoctavirus* members.

### 2.11. Antibiofilm Activity of Phage vKPPW7

**Figure 7:**
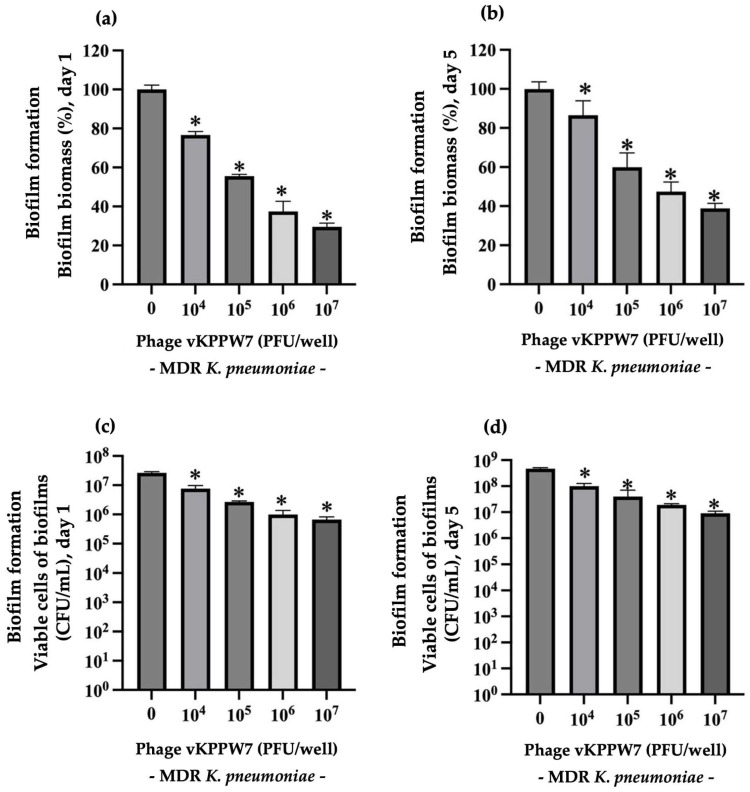
Ability of phage vKPPW7 to reduce biofilm formation. (**a**) The effect of phage vKPPW7 on the biomass of biofilm formation in 1-day-old biofilms; (**b**) The effect of phage vKPPW7 on the biomass of biofilm formation at 5-day-old biofilms; (**c**) The effect of phage vKPPW7 on the biofilm cell viability in 1-day-old biofilms; (**d**) Effect of phage vKPPW7 on the biofilm cell viability in 5-day-old biofilms. The bars represent the SEM, and asterisks denote statistically significant differences (* *p* ≤ 0.05).

The effectiveness of phage vKPPW7 in preventing biofilm formation was evaluated at 1 day post-incubation (young biofilms) and 5 days post-incubation (mature biofilms). Biofilm biomass and cell viability were assessed using crystal violet staining and quantification of colony-forming units (CFUs) on separated plates. To evaluate the ability of phage vKPPW7 to prevent biofilm formation, MDR *K. pneumoniae* isolates were treated with varying concentrations of phage vKPPW7. After 1 day of incubation, the biofilm biomass significantly decreased by approximately 23.36 to 70.47% after incubation with the phage at 10^4^ to 10^7^ PFU/well compared to the control, with this effect observed in a dose-dependent manner (Figure 7a). Additionally, treatment with the phage at 10^4^ to 10^7^ PFU/well resulted in a reduction in biofilm bacterial viability by 0.49 to 1.83 log compared to the control, respectively (Figure 7c). At *5* days post-incubation, phage vKPPW7 led to a significant reduction in biofilm biomass by approximately 13.41 to 67.18% (Figure 7b). Bacterial cell viability was reduced by 0.57 to 1.57 log following treatment with the phage at 10^4^ to 10^7^ PFU/well (Figure 7d).

**Figure 8:**
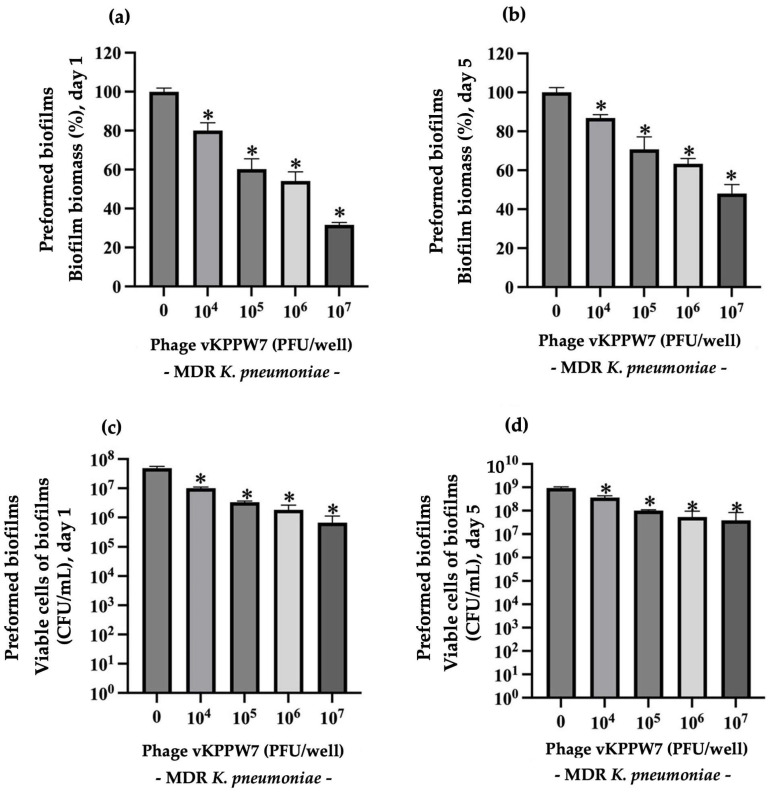
Ability of phage vKPPW7 to remove preformed biofilms. (**a**) The effect of phage vKPPW7 on the biomass of 1-day-old preformed biofilms; (**b**) The effect of phage vKPPW7 on the biomass of 5-day-old preformed biofilms; (**c**) The effect of phage vKPPW7 on the biofilm cell viability within 1-day-old preformed biofilms; (**d**) Effect of phage vKPPW7 on the biofilm cell viability within 5-day-old preformed biofilms. The bars represent the SEM, and asterisks denote statistically significant differences (* *p* ≤ 0.05).

Preformed biofilms of MDR *K. pneumoniae* were cultured for 1 day (young biofilms) and 5 days (mature biofilms), followed by treatment with phage vKPPW7. For 1 days old preformed biofilms, phage concentrations ranging from 10^4^ to 10^7^ PFU/well significantly reduced preformed biofilm biomass by an average of 19.96 to 68.30%, compared to the control (Figure 8a). Similarly, the viability of biofilm bacteria was significantly reduced by approximately 0.49 to 1.82 log, compared to that of the control (Figure 8c). Treatment of 5 days old preformed biofilms with phage concentration ranging from 10^4^ to 10^7^ PFU/well resulted in the eradication of 13.17 to 51.97% of biofilm biomass (Figure 8b) and a reduction of 0.47 to 1.55 log in bacterial cell viability compared to the control (Figure 8d).

### 2.12. Visualization of Biofilms After Phage vKPPW7 Treatment Using SEM

**Figure 9:**
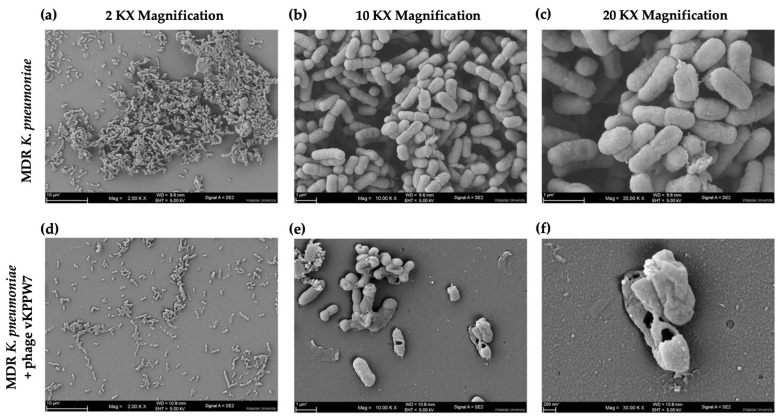
Observation of biofilm structures using SEM. (**a**–**c**) Five-day-old biofilms of MDR *K. pneumoniae*; (**d**–**f**) Five-day-old biofilms of MDR *K. pneumoniae* treated with phage vKPPW7. Figures (**a**) and (**d**) were captured at 2000× magnification, while figures (**b**) and (**e**) were captured at 10,000× magnification, and figures c and f were captured at 20,000× magnification.

The architecture of biofilms, both with or without phage vKPPW7 treatments, was visualized under SEM. Biofilms were grown for 5 days and then treated with phage vKPPW7. Image analysis and bacterial quantification were performed using ImageJ (version 1.54g). Representative SEM micrographs of biofilms produced by MDR *K. pneumoniae*, as a control, are shown in Figure 9a–c). A significant amount of biofilm was formed on the coverslip, with bacterial aggregations in a three-dimensional structure and a high level of extracellular matrix. The morphology of bacterial cells in biofilms appeared normal, with a smooth surface. In images captured at 10 kx magnification, the control group exhibited an average bacterial count of 176.33 ± 21.03 cells.

In contrast, the amount of biofilm treated with phage vKPPW7 was reduced compared to the untreated control (Figure 9d–f). Only a small number of bacterial cells adhered to the coverslip in the treated group, with an average count of 28.67 ± 9.27 cells. Moreover, all observed bacterial cells were damaged cells, exhibiting pores, and blebbing membranes, indicative of bacterial cell death. The level of extracellular matrix was lower than in the treated biofilms compared to the control.

## 3. Discussion

Antibiotic-resistant *K. pneumoniae* is one of the most critical nosocomial pathogens, impacting both humans and animals. In recent years, MDR *K. pneumoniae* infections have emerged as a significant global public health concern [23,24]. Bacterial resistance to fluoroquinolones and β-lactam antibiotics, encompassing carbapenems, cephalosporins, and penicillin, is responsible for over 70% of deaths attributable to antimicrobial resistance [25]. Nowadays, this bacterium exhibits resistance to last-resort options such as carbapenems and colistin. Alarmingly, *K. pneumoniae* resistance rates have increased, reaching 100% resistance in certain regions of Saudi Arabia [26]. Consequently, exploring complementary and alternative medicines is imperative in combating MDR *K. pneumoniae*.

In this study we describe a novel phage specific to MDR *K. pneumoniae* with capsular serotype K24 designated as phage vKPPW7. Plaque formation with halo zones on the lawn of the MDR *K. pneumoniae* indicated the presence of a polysaccharide depolymerase enzyme encoded by the phage. Morphological analysis of phage particles under TEM revealed *Podoviridae* morphology. Whole-genome sequencing confirmed the phage classification. The evaluation of the phage ability to target MDR *K. pneumoniae* isolates demonstrated its potential as an alternative treatment for infections caused by these resistant bacteria. The high adsorption rate of the phage is advantageous for efficient attachment [27]. Phage vKPPW7 exhibited rapid adsorption into bacterial cells. In comparison, phage KP1LMA showed a slower adsorption rate, with 75% of the phage particles adsorbed to *K. pneumoniae* Scc 24 after 60 min. Conversely, phage PG14 achieved 90% adsorption on its host within 12 min [28,29]. The results indicate its efficient attachment to host bacterial cells. However, the adsorption rate may vary depending on environmental factors such as cations, pH, and temperature [30]. Phage vKPPW7 remained stable over a wide range of temperatures and pHs. A comparative analysis of latent periods and burst sizes with other *Klebsiella* phages revealed that phage vKPPW7 exhibits a short latent period (20 min) and large burst size (106 PFU/cell). For example, Phage JKP2, which targets the *Klebsiella* K17 capsular serotype, has a latent period of 45 min and produces a burst size of 70 PFU/cell [31]. The strong bactericidal activity of phage vKPPW7 was clearly observed in killing kinetics studies. Although the growth of phage-resistant bacteria in the phage-infected bacteria with low MOI slightly increased after 8 h of phage incubation, a higher MOI of phage was still able to suppress bacterial growth completely. Considering all these biological characteristics, our study suggests phage vKPPW7 as a promising candidate for various phage applications.

The whole genome of phage vKPPW7 was studied, and the results suggest the novelty of phage vKPPW7 within the genus *Kaypoctavirus* and its classification within the subfamily *Enquatrovirinae*, family *Schitoviridae*, class *Caudoviricetes*, phylum *Uroviricota*, kingdom *Heunggongvirae*, and realm *Duplodnaviria*. The absence of antibiotic-resistance genes, virulence-associated genes, or lysogeny-related genes in the genome suggests that the phage may be a good candidate for phage therapy. The genetic relationship analysis confirmed that phage vKPPW7 is closely related to other phages in the genus *Kaypoctavirus*.

Interestingly, phage-encoded proteins, particularly the tail fiber protein, and the halo zone of plaques, which is indicative of phage-encoded depolymerase enzyme activity, may contribute to anti-biofilm activities, suggesting potential antibiofilm activity of phage vKPPW7 [32,33]. Biofilm formation, prevalent in *K. pneumoniae* infection, poses significant challenges due to its role in antibiotic resistance [34,35]. Extracellular polymeric substances (EPS) can decrease or prevent antibiotic penetration [36]. Phages can disrupt biofilms through various mechanisms by targeting specific bacterial cells [37,38]. One of the most important mechanisms involves the degradation of biofilms by phage enzymes, such as depolymerase and endolysin enzymes [39,40]. Depolymerase enzymes, encoded by phage tail spike proteins, recognize and bind to specific bacterial cells, leading to the disruption of EPSs formed by bacterial cells. Subsequently, phages can degrade the biofilm structure, followed by penetration into the inner biofilm layers [37,41]. In addition, endolysins or phage lysins have been reported to possess biofilm removal capabilities. Interactions between endolysin and EPS may influence the adhesion of competing viruses, the availability of phage receptors, and the formation of cell wall-deficient cells [42]. The identification of a depolymerase domain in the genome of phage vKPPW7 using the Phyre 2 server suggests its potential for antibiofilm activity. Thus, our study aimed to investigate the ability of phage vKPPW7 to prevent and eradicate biofilms. The efficacy of phage vKPPW7 to control mature biofilms (defined as 5-day old in this study) was also interrogated, as it has been shown that mature biofilms of *E. coli* can impede phage diffusion and phage release [43,44], as well as affect phage penetration and proliferation [45]. Moreover, mature biofilms are associated with chronic infections that persist over long periods of time [46]. The results demonstrated that phage vKPPW7 could effectively remove both young and mature biofilms. Moreover, phage vKPPW7 could prevent biofilm formation. Phage-encoded depolymerase enzymes disrupted the biofilm structure by digesting EPS, while phage-mediated killing of planktonic bacteria further inhibited biofilm formation [41,47]. Phage might reduce the migration of bacteria, preventing further diffusion of biofilms [48]. The results of the phage biofilm challenge assays demonstrate a strong correlation between both biomass reduction and cell viability and the concentrations of phage vKPPW7.

## 4. Materials and Methods

### 4.1. Bacterial Strains and Growth Conditions

Eighteen *K. pneumoniae* clinical isolates with capsular type K24 were sourced from bacterial stocks in the laboratory. These isolates originated from routine laboratory specimens collected at Songklanagarind Hospital, Prince of Songkla University, in Songkhla Province, Thailand. Additionally, 2 clinical isolates of *K. pneumoniae* with non-capsular type K24, *A*. *baumannii* ATCC 17978, 1 clinical isolate of *E. coli* ECPW01, and 1 clinical isolate of MRSA PW01 were included in this study. Bacterial colonies were picked from a tryptic soy agar (TSA; Becton, Dickinson and Company, Franklin Lakes, NJ, USA) plate and transferred to a tube containing 3 mL of tryptic soy broth (TSB; Becton, Dickinson and Company, Franklin Lakes, NJ, USA). The cultures were then incubated at 37 °C with continuous shaking at 150 rpm for either 6 h or overnight. Before the experiment began, bacteria were diluted in TSB to achieve the desired colony-forming units per milliliter (CFU/mL) value.

### 4.2. Antimicrobial Susceptibility Test

The antimicrobial susceptibility profiles of *K. pneumoniae* were determined using the disc diffusion method, following the Clinical and Laboratory Standards Institute guidelines [49]. Antibiotic discs (Oxoid^TM^, Thermo Fisher Scientific Inc., Waltham, MA, USA) containing AMP (10 µg), CAZ (30 µg), CIP (5 µg), CTX (30 µg), GEN (10 µg), IMP (10 µg), and MER (10 µg) were employed in this study.

### 4.3. Phage Enrichment and Isolation

Environmental water samples collected from Thasala, Nakhon Si Thammarat, Thailand, were used for the phage isolation. MDR *K. pneumoniae* clinical isolate KPPW67 served as the bacterial host for this process. Briefly, water samples were centrifuged to remove debris at 6000× *g*, 4 °C for 10 min. The supernatant was filtered through a sterile 0.22 μm filter (Merck Millipore, Burlington, MA, USA), and 10 mL of the filtrate was then inoculated with 10 mL of TSB and 200 μL of exponential-phase MDR *K. pneumoniae*. Following overnight incubation at 150 rpm at 37 °C, the culture was centrifuged to remove bacterial cells at 6000× *g* for 20 min at 4 °C and then filtered through a sterile 0.22 μm filter. The collected filtrate was then used for phage detection by the double-layer agar method.

### 4.4. Double-Agar Overlay Method

A double-agar overlay method was employed for phage isolation and purification as described previously [50]. The samples were serially diluted 10-fold with sodium chloride–magnesium sulfate (SM) buffer (50 mM Tris-HCl (pH 7.5), 0.1 M NaCl, and 8 mM MgSO_4_·7H_2_O). Log-phase bacteria were diluted to an OD_600_ of 0.1. Top agar (0.75% agar in TSB) was supplemented with 200 μL of the phage solution and 200 µL of the diluted bacteria, and the mixture was immediately poured onto TSA plates. The plates were then incubated overnight at 37 °C to observe the formation of plaques.

### 4.5. Phage Purification and Amplification

The individual plaques were picked from the lawn of host bacteria and transferred into an Eppendorf tube containing 500 μL of SM buffer. Following overnight incubation at 4 °C, the samples were centrifuged at 6000× *g* for 10 min at 4 °C. The supernatant was collected and filtered through a sterile 0.22 μm filter. The filtrate was then serially diluted in SM buffer and used in the double-agar overlay method. This purification process was repeated three times.

The double-agar overlay method was used to obtain phage stocks. Semi-confluent plates were selected for phage stock collection. Each plate was filled with SM buffer and incubated at 4 °C overnight. After collecting the buffer, bacterial debris was removed by centrifugation at 6000× *g* for 20 min at 4 °C. The phage lysate was then purified by filtration using a sterile 0.22 μm syringe filter. Phage titer was determined by the double-agar layer method, with the unit expressed as PFU/mL.

### 4.6. Microscopy Observation of Phage Virions

TEM was used to characterize virion morphology. Twenty microliters of the phage stock were deposited onto carbon-coated copper grids, which were subsequently stained with 2% (*v*/*v*) uranyl acetate (pH 6.7). The phage morphology was then examined using a JEM 2010 electron microscope (JEOL, Freising, Germany) at an operating voltage of 160 kV.

### 4.7. Determination of Phage Host Range

To screen the ability of phage vKPPW7 to lyse bacterial isolates, 18 *K. pneumoniae* clinical isolates with capsular type K24, 2 clinical isolate of *K. pneumoniae* with non-capsular type K24, *A*. *baumannii* ATCC 17978, 1 clinical isolate of *E. coli* ECPW01 and 1 clinical isolate of MRSA PW01 were tested using the standard spot test method [50]. The top agar was supplemented with 200 μL of exponential-phase bacteria and poured onto a TSA plate. Ten microliters of the phage suspension at 10^4^ PFU/mL was dispensed onto the bacterial lawn and allowed to air-dry. The plates were subsequently incubated overnight at 37 °C. The presence of a lysis zone was noted as “+” while its absence was marked as “−”. Each experiment was performed in triplicate and repeated twice.

### 4.8. EOP

To assess the lytic activity of the phage, EOP was carried out as previously described [51]. The sensitive bacterial isolates in the host range assay and the bacterial host isolate were used to study EOP. Each log-phase bacterial isolate (200 µL) was mixed with 200 μL of serially diluted phage solution at an MOI of 0.01. After a 15 min incubation at room temperature, the mixtures were assessed using the double-agar overlay method. EOP was determined by calculating the ratio of the average PFU on the tested isolate to the average PFU on the isolate with the maximum plaque counts (the host bacterium). Experiments were conducted independently in triplicate, with each set including duplicate plaque assays, and the entire process was repeated twice. EOP values were categorized as “high efficiency” (EOP ≥ 0.5), “medium efficiency” (0.1 ≤ EOP < 0.5), “low efficiency” (0.001 < EOP < 0.1), or “inefficient” (EOP ≤ 0.001).

### 4.9. Stability of Phage vKPPW7 at Different Temperature and pH Ranges

The stability of the phage was assessed at various temperatures and pHs using a previously described method [50,52]. The phage was initially diluted in SM buffer to achieve a concentration of 10^8^ PFU/mL and then incubated at different temperatures (4 °C, 25 °C, 37 °C, 50 °C, 60 °C, 70 °C and 80 °C) for 2 h, with 4 °C serving as the control. Subsequently, the phage was diluted, and its stability was determined using the double-agar overlay method.

To investigate the influence of pH on phage stability, 100 μL of phage vKPPW7 was mixed with SM buffer adjusted to various pH values ranging from 1 to 14 for 2 h, and the final concentration of the phage was maintained at 10^8^ PFU/mL. Following incubation, the pH of phage suspension was neutralized to pH 7, and phage stability was determined using the double-layer agar method. Phage samples maintained at pH 7 served as controls. Experiments were conducted independently in triplicate, with each set including duplicate plaque assays, and the entire process was repeated twice.

### 4.10. Phage Adsorption Test

The kinetics of phage adsorption were conducted following previously established methods [53,54]. MDR *K. pneumoniae* bacteria were inoculated with the phage at an MOI of 1 and then incubated at 37 °C without shaking. Samples were collected and immediately processed through a sterile 0.22 μm syringe filter at one-minute intervals for a total of 10 min. The phage titer in both the supernatant and pellet was measured using the double-agar layer method. The adsorption rate constant for phage attachment to the host cell was calculated and expressed as mL/min. All experiments were performed in triplicate, with each set of triplicates subjected to duplicate plaque assays. Experiments were repeated twice.

### 4.11. One-Step Growth Curve

A one-step growth curve was performed to evaluate the infectivity and replication ability of the phage, as described previously [12,55]. Phage at an MOI of 0.1 was mixed with exponential-phase MDR *K. pneumoniae* cells and incubated for 10 min. To remove unabsorbed phages, the samples were centrifuged at 6000× *g* for 20 min at 4 °C. The supernatant was discarded, and the pellet was resuspended in TSB. The phage suspension was then incubated at 37 °C with shaking at 150 rpm. During the 120 min incubation, the samples were collected every 10 min for phage titer determination using the double-layer agar method. The latent period (time between absorption and the first burst) and burst size (the ratio of the final number of increased phages to the initial number of bacterial cells) were calculated. Independent experiments were conducted in triplicate, with each experiment including duplicate plaque assays, and the entire process was repeated twice.

### 4.12. In Vitro Lytic Activity of Phage Against MDR K. pneumoniae

The in vitro lytic activity of the phage in a liquid environment was assessed against MDR *K. pneumoniae*. Briefly, MDR *K. pneumoniae* were infected with the phage at various MOIs of 0.01, 0.1, and 1, followed by incubation in a shaking incubator at 37 °C at 150 rpm. Samples were collected hourly over a 10 h period, and the optical density at 600 nm was measured using a UV-spectrophotometer. MDR *K. pneumoniae* cultures without phage infection served as the control sample. Experiments were conducted independently in triplicate and repeated twice.

### 4.13. Morphological Analysis of the Phage-Infected Bacterial Cells by SEM

SEM was used to examine the morphology of MDR *K. pneumoniae* cells after treatment with phage vKPPW7 at an MOI of 0.1. To prepare the samples, bacterial cells were mixed with the phage and incubated at 37 °C for 2 h. The mixture was then centrifuged at 6000× *g* for 5 min to pellet the cells, and the supernatant was discarded. The pellet was washed three times with PBS to remove the residual medium. Subsequently, the bacterial cells were deposited onto coverslips and fixed by incubation with 2.5% glutaraldehyde in 0.1 M PBS at 4 °C overnight. After fixation, the cells were washed three times with 0.1 M PBS and further fixed with 1% Osmium tetroxide (OsO_4_) in DI water for 1 h. The bacterial cells were subsequently washed twice with PBS, and dehydration was carried out using a graded ethanol series (20%, 40%, 60%, 80%, and 100%). Following dehydration, the cells were subjected to critical point drying (CPD). The prepared samples were then coated with gold, and the bacterial morphology was observed using a field-emission SEM (Zeiss, Oberkochen, Germany).

### 4.14. Whole Genome Sequencing and Bioinformatic Analysis

Phage genomic DNA was extracted using a phage DNA isolation kit (Norgen Biotek Corp., Thorold, ON, Canada) according to the manufacturer protocol. The extracted DNA was assessed for both qualitative and quantitative integrity. Subsequently, the phage genomic DNA was fragmented, and the sequencing libraries were prepared using the TruSeq Nano DNA library preparation kit (Illumina Inc., San Diego, CA, USA). The library preparation involved random fragmentation of DNA followed by ligation of 5′ and 3′ adaptors. Adapter-ligated fragments were amplified by PCR, and the quality of the libraries was evaluated using an Agilent Technologies 2100 Bioanalyzer. Sequencing was conducted using the Illumina sequencing platform (Macrogen Inc., Seoul, Republic of Korea), and the quality of the raw sequencing data was assessed using FastQC (version 0.11.5). Filtered high-quality data were assembled de novo into a contig using SPAdes (version 3.15.0) with a k-mer size of 123 bp. The de novo assembly was followed by annotation of genes, construction of the genome map, and prediction of tRNA genes, tmRNA genes, and CRISPR elements using the Pharokka program (version 1.3.0) [56]. Gene annotation was also analyzed using the PHASTEST web server [57].

The phylogenetic tree was constructed by the ViPTree web server. The genomic comparison of phage vKPPW7 and its closest relative was also performed with the same tool. The online EMBOSS Stretcher analysis web server was utilized to analyze the new species of the phage with a pair parameter by comparing genomic synteny and sequence identity [58]. The depolymerase domain in the tail fiber protein was predicted using the PHYRE automatic fold recognition server [59].

### 4.15. Phylogenetic Analysis of Specific Genes

The large terminase and capsid were employed as models for constructing phylogenetic trees. Amino acid sequences of each protein were queried against the NCBI databases using BlastP, with an E-value cutoff of 0, a query coverage cutoff of 100%, and an identity cutoff of 85%. Subsequently, amino acid sequences of the selected phage proteins were retrieved from the database. Genetic relationships between phage vKPPW7 and the selected phages were analyzed using a maximum-likelihood phylogenetic tree and JTT matrix-based model, based on amino acid sequence alignment in MEGA-X.

### 4.16. Efficacy of Phage in Preventing Biofilm Formation

The ability of phage vKPPW7 to inhibit biofilm formation was evaluated by measuring biofilm biomass and viable cell counts using established protocols [12,60]. In brief, MDR *K. pneumoniae* KPPW67, the host bacterium for phage vKPPW7, was introduced into flat-bottomed 96-well microtiter plates. Subsequently, 100 μL of phage vKPPW7 was added into each well, with the final concentrations ranging from 10^4^ to 10^7^ PFU/well. The plates were incubated at 37 °C without agitation. At designated time points (1 and 5 days), the medium containing planktonic bacteria was carefully removed. Non-adherent cells and debris were eliminated by washing the wells twice with pre-warmed PBS.

To determine biofilm biomass, the plates were air-dried and then incubated with 200 μL of 0.1% crystal violet for 30 min. After removing the crystal violet staining solution, the wells were washed three times with PBS to eliminate excess stain. The biofilms were solubilized with 200 μL of absolute ethanol and then measured at the OD595 using a standard microplate absorbance reader. To assess viable cell counts, 200 µL of TSB was added to the wells to resuspend the biofilms. The solution was serially diluted and then plated onto TSA plates. After overnight incubation at 37 °C, bacterial colonies were counted. Experiments were conducted independently in triplicate and repeated twice.

### 4.17. Biofilm Eradication of the Phage

The ability of the phage to eradicate preformed biofilms was assessed [12]. The phage host strain, MDR *K. pneumoniae* KPPW67, was cultured in a new flat-bottomed 96-well microtiter plate for 1 and 5 days. To establish a 5-day-old biofilm, spent media were replaced with fresh TSB every day until the fifth day of the experiment. The media were removed at the indicated time points, and the wells were washed twice with pre-warmed PBS. One hundred microliters of the indicated phage concentrations (ranging from 10^4^ to 10^7^ PFU/well) was inoculated to the wells, followed by the addition of 100 µL of TSB. The plates were then incubated at 37 °C for 24 h without agitation. Subsequently, as described above, the biofilm biomass and viable cells were assessed using crystal violet staining and viable cell counts, respectively.

### 4.18. Biofilm Formation Assay for SEM

Preformed biofilms on day 5 were selected as the model. MDR *K. pneumoniae* was cultured in 24-well plates containing glass slides. The plates were then incubated at 37 °C in the incubator, with spent media being replaced with fresh TSB daily until the fifth day of the experiment. One milliliter of phage vKPPW7 at a 10^7^ PFU/mL concentration was added to each well and then incubated at 37 °C for 24 h. MDR *K. pneumoniae* without phage treatment served as the control. Subsequently, the wells were washed three times with PBS, and the coverslips were fixed with 2.5% glutaraldehyde, followed by fixation with 1% OsO_4_ solution. The samples were dehydrated in an ethanol-water mixture with increasing ethanol concentrations. Finally, the coverslips were rehydrated with CPD, coated with gold, and observed under SEM as described above.

### 4.19. Statistical Analyses

All data was analyzed using the GraphPad Prism program, version 10 (GrapPad Software, https://www.graphpad.com/scientific-software/prism/, accessed on 29 September 2024). Statistical significance was determined by an unpaired *t*-test using the GraphPad Prism, with *p*  <  0.05 considered significant.

### 4.20. Nucleotide Sequence Accession Numbers

The complete genome sequence was deposited in the GenBank database under the accession number PQ409239.

## 5. Conclusions

Due to the efficacy of phage vKPPW7 in killing MDR *K. pneumoniae* with capsular serotype K24, our results suggest its potential as both an effective antibacterial agent and a promising tool for biofilm control. The study positions phage vKPPW7 as a promising candidate for further exploration in phage therapy, particularly due to its high adsorption rate, short latent period, large burst size, and capacity to prevent and eradicate biofilms. Developing phage vKPPW7 specific to *K. pneumoniae* with capsular serotype K24, along with other phages targeting *K. pneumoniae* with non-capsular serotype K24, into a phage cocktail could be beneficial, as it may effectively combat diverse K types and limit the bacterial evolution of phage resistance. This research contributes to the growing body of evidence supporting the potential of phage therapy in treating bacterial pathogens, especially in biofilm-related infections.

## Data Availability

The original contributions presented in this study are included in the article/Supplementary Material. Further inquiries can be directed to the corresponding author.

## Supplementary Materials

The following are available online at https://www.mdpi.com/article/10.3390/antibiotics14020157/s1, Table S1: Antimicrobial susceptibility profiles of MDR *K. pneumoniae*, Table S2: Bacterial lysis efficacy and EOP of phage vKPPW7, Table S3: Whole genome analysis of phage vKPPW7 by the Pharokka program, Table S4: Whole genome analysis of phage vKPPW7 by FASTEST.
